# On-Site Earthquake Early Warning Using Smartphones

**DOI:** 10.3390/s20102928

**Published:** 2020-05-21

**Authors:** Ting-Yu Hsu, C. P. Nieh

**Affiliations:** Department of Civil and Construction Engineering, National Taiwan University of Science and Technology, Taipei 10607, Taiwan; karta1649762@hotmail.com

**Keywords:** earthquake early warning, on-site, smartphone, artificial neural network, peak ground acceleration

## Abstract

In this study, the measured accelerations of a single smartphone were used to provide an earthquake early warning system. In the presented system, after the smartphone is triggered, the triggering event is then classified as an earthquake event or not. Once an earthquake event is detected, the peak ground acceleration is then predicted every second until 10 s after the trigger. These predictions are made by the neural network classifier and predictor embedded in the smartphone, and an alert can be issued if a large peak ground acceleration is predicted. The proposed system is unique among approaches that use crowdsourcing ideas for earthquake early warning because the proposed system provides on-site earthquake early warning. In general, the accuracy rates of the earthquake classifications and peak ground acceleration predictions of the system were quite high according to the results of large amounts of earthquake and non-earthquake data. More specifically, according to said earthquake data, 96.9% of the issued alerts would be correct and 61.9% of the earthquakes that exceeded the threshold would have resulted in an alert being issued before the arrival of the peak ground acceleration. Among the false negative cases, approximate 97.8% would occur because of negative lead time. Using the shake table tests of worldwide and Meinong earthquake datasets, the proposed approach is confirmed to be quite promising.

## 1. Introduction

Earthquake early warning (EEW) aims to deliver alerts to the public and automated systems before destructive waves strike them. Although the idea was first proposed by Cooper in the mid-19th century [1], EEW systems have only been realized in recent decades thanks to advances in communications, digital seismology, and automatic processing [2]. The development and implementation of EEW systems were summarized in previous studies by Allen et al. [3] and Allen and Melgar [4], and it is still an emerging research topic in the community [5,6,7,8,9]. The EEW systems could also incorporate with structural control algorithms for better seismic response reduction [10,11]. However, the application of EEW systems has been limited to those regions with seismic networks, which are costly in terms of both installation and operation.

EEW systems can mainly be divided into two categories, namely regional and on-site ones. A regional EEW system requires a seismic network, while on-site EEW systems only use one seismic station. Recently, the idea of participatory sensing, in which mobile devices are used collectively to gather critical information, was introduced by Burke et al. [12]. With respect to EEW, Minson et al. [13] have demonstrated that regional EEW systems could be achieved via crowdsourced smartphone-based approaches. They indicated that when a seismic motion is sufficiently large, the global positioning system sensor on a smartphone can be used to detect ground motion.

The Earthquake Network project [14,15] developed a crowdsourced regional EEW system based on a smartphone network. This system is based on the assumption that when earthquake waves hit an area with a sufficiently large population, a far greater number of smartphones would be shaken than would be shaken during non-earthquake times. The system then utilizes the spatial distribution of the reporting smartphones and a statistical approach to detect changes from normal background levels of shaking reports and identify a rough epicenter location for the earthquake in question.

The MyShake app [16,17] offers another option for turning smartphones into seismometer networks to provide regional EEW. It also exploits the computational resources of smartphones to distinguish whether the movements of those smartphones are caused by earthquakes or human activities. The location, origin time, and magnitude of any detected earthquakes are then estimated by a real-time server using the earthquake triggers uploaded from the network phones. Until now, only the Earthquake Network and MyShake apps used smartphone accelerometers to detect earthquake ground shaking.

**Figure 1:**
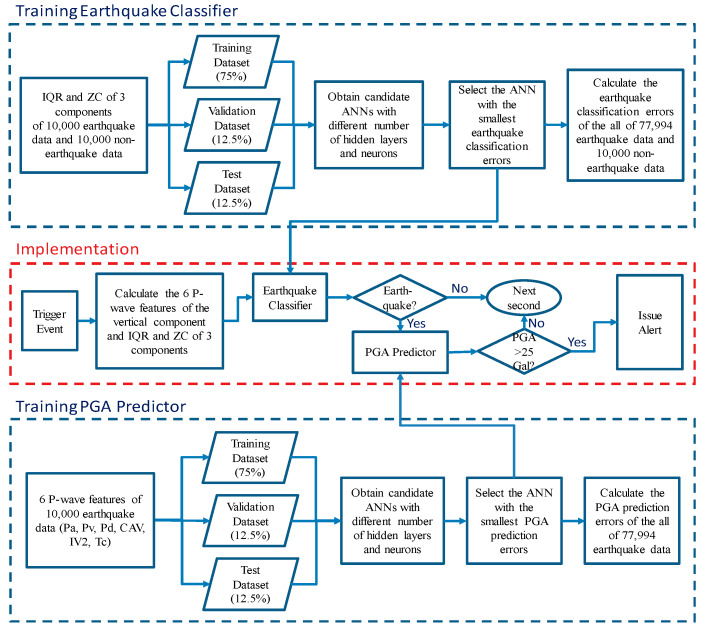
The flow chart of training the earthquake classifier, training the PGA predictor, and implementation of these two models.

In this study, we propose a system that further leverages smartphone computational resources to run a single-station (on-site) EEW algorithm in each smartphone. For this system, the artificial neural network (ANN) approach was used to construct a classifier for distinguishing earthquakes from human activities, and also to construct a peak ground acceleration (PGA) predictor based on the accelerations measured by smartphones. The flowchart of training the earthquake classifier, training the PGA predictor, and implementation of these two models is summarized in Figure 1. The data used to train, validate, and test the classifier and predictor are described in the second section of this paper. The procedure and results of the earthquake classification and PGA prediction of the system are detailed in the third and fourth section, respectively. The performance of the proposed system in terms of issuing alerts regarding earthquakes is discussed in the fifth section. The further verification of the proposed approach using shake table tests is described in the sixth section. Finally, the conclusion and related future projects are discussed in the final section of this paper.

## 2. Smartphones and Earthquake Datasets

Previous studies have confirmed that smartphones fixed on shake tables can measure ground motion accurately between 0.5 and 10 Hz [18,19]. When the horizontal accelerations of a sweep excitation reach approximately 0.3 g and above 3 Hz [17], the sliding of smartphones can be observed during the excitation, and the effect of clipping the peak amplitudes starts. As a result, the recorded time history is distorted. For the application in this study, only the first several seconds after the triggering of the smartphones was used for analysis. The short-term average/long-term average (STA/LTA) algorithm was applied to automatically determine the *P*-wave arrival time. In general, the amplitude during the first several seconds after a trigger is quite small; hence, using smartphones for on-site EEW is valid in most cases. The exceptions in which the amplitude is large during the first several seconds after a trigger occur when smartphones are close to the epicenter of an earthquake. In such cases, however, the smartphones are very possibly within the regions of the blind zone for EEW in which no alerts can be issued before the strong waves arrive.

In this study, five mid-priced smartphones of different brands were used, as shown in Table 1. The resolution of the measured accelerations of each smartphone is also listed in the same table. Noise floor tests were performed to estimate the noise floors of the smartphones by calculating the root mean square of the accelerations measured when the smartphones were placed in the quiet basement of a building, and the noise floors are also listed in Table 1.

In order to develop the algorithms used to distinguish whether the movements of smartphones have been caused by earthquakes or human activities, the accelerations measured by smartphones resulting from both human activities and earthquakes are required. In this study, human activity data were collected from the smartphones as the smartphones remained stationary for at least 10 min. Once the ratio of the short-term average and long-term average on any of the three components exceeded a threshold [20], the smartphone was triggered and the acceleration data from one minute before the trigger to four minutes after was recorded. In total, 49,094 triggers were recorded during the period.

The earthquake data used in this study were replicated from existing ground motions recorded by the Taiwan Strong Motion Instrument Program (TSMIP) maintained by the Central Weather Bureau (CWB) in Taiwan. In order to generate acceleration signals similar to what could be measured on steady smartphones during real earthquakes, the ground motions were converted to the resolution of each smartphone, and then the noise signals of each smartphone recorded during the noise floor tests were added to the ground motions. The TSMIP data recorded from 29th July 1992 to 31st December 2006 were used in this study. Because the noise floor and resolution of each smartphone are different, the number of ground motions produced for each smartphone were different, as shown in Table 1. The number of ground motions for most of the smartphones was more than 60,000, while that for the ASUS ZenFone Selfie was only 36,476, mainly because its noise floor is higher than those of the other smartphones used.

## 3. Earthquake Detection

Kong et al. [17] developed a classifier to detect earthquakes on a smartphone. They proposed the use of an ANN approach to classify whether or not the data in a 2 s data window resulted from an earthquake. In other words, only the 2 s windows of data with the largest recorded ground motion amplitudes were used as the earthquake data to train the ANN classifier. They suggested that the best three features of such 2 s data windows for distinguishing between earthquakes and human activities are the interquartile range (IQR) between the 25th and 75th percentile of the acceleration vector sum of the three-component acceleration, the zero crossing rate from the component with the highest value (ZC), and the cumulative absolute velocity (CAV) of the acceleration vector sum of the 3-component acceleration. In their study, an ANN with one hidden layer of five neurons with sigmoid activation function and one output was used.

In this study, 75%, 12.5%, and 12.5% of both 10,000 pieces of earthquake data and 10,000 pieces of non-earthquake data were used to train, validate, and test the ANN classifier, respectively. The non-earthquake data were randomly selected from the available database. As for the earthquake data, we tried to keep the numbers of selected pieces of data with different PGA ranges as even as possible. We selected the LG Stylus smartphone, which had the worst resolution and moderate noise floor among the available smartphones, as a representative benchmark. The results of all the available earthquake and non-earthquake data are shown in the following paragraphs.

First, we use the features of the first two seconds after a trigger of both earthquake and non-earthquake data as the inputs of the ANN. The features we used are the IQR and ZC of three components (six features in total). The CAV was not used because we found that no significantly better results were obtained if it was included. A grid search was conducted to test different numbers of neurons and layers. The best performance was achieved when the ANN had two hidden layers with 15 neurons with sigmoid activation function. The true positive rate (TPR; that is, when an event classified as an earthquake actually was an earthquake) and true negative rate (TNR; that is, when an event classified as a non-earthquake actually was not an earthquake) of the ANN classifier when the data from the first 2 s after a trigger were used were 99.09% and 97.85%, respectively.

After the 2 s ANN classifier was obtained, ANN classifiers using the features of N seconds after a trigger were constructed in a similar way, with N starting between one second to ten seconds after the trigger. The features of the first N seconds after a trigger of both earthquake and non-earthquake data were used as the inputs of the ANN. For each length of N, an ANN classifier was trained. Therefore, a total of 10 ANN classifiers were obtained. In order to reduce the possibility of missing an earthquake, the trigger event was classified as an earthquake once it was classified as an earthquake at any second after the trigger using the corresponding ANN classifier. The number of cases missed (earthquakes classified as non-earthquakes) as a result among all 77,994 cases of earthquake data with different PGA ranges after N seconds are listed in Table 2. At the first second, only 1350 cases in total (approximately 1.7%) were missed, and the PGA values of some of them were larger than 80 Gals. The number of missed cases then decreased rapidly as the length of time after the trigger was increased. After five seconds, only 12 cases were missed (0.015%), and the PGA values of all the missed cases were smaller than 80 Gals. These results imply that almost all the large earthquakes would be detected successfully. On the other hand, the possibility that non-earthquake cases are classified as earthquake cases also increased as the length of time after the trigger was increased. The false positive ratios (FPR; that is, when an event classified as an earthquake was not actually an earthquake) of all the 49,094 non-earthquake cases are also listed in Table 2. The FPR at the first second was only 3.20%, but increased to 6.76% at the 10th second. For reference, the FPR of the ANN algorithm of the MyShake app was also reported to be approximately 7% [17]. In order to prevent a high possibility of false alerts, a network detection algorithm can be used. For example, only when a high percentage of operating smartphones within a certain distance range of the location of the event are classified as reporting earthquakes, will an alert be announced from the server to the smartphone users. However, in this study, we only focused on the development of an on-site EEW application for a single smartphone. A network detection function will be presented in a future related study.

The above classifier was developed for the LG Stylus smartphone, i.e., it was the LG classifier model. If this model was applied to the other four smartphone models without any adjustment, the performance would be quite bad, mainly because the resolution of the LG Stylus smartphone was quite large relative to those of the other smartphone models. However, when the accelerations of these four smartphones were converted to the resolution of the LG Stylus smartphone, the performance of the smartphones when using the LG classifier model was quite good. The typical TPR and TNR results of the 2 s ANN classifier of each smartphone are summarized in Table 3. Note that the TPRs of all the smartphones were quite high, ranging from 98.51% to 99.70%. The TNRs of the Redmi Note4 and Huawei P3 were also quite high. However, the TNR of the HTC Desire Eye was slightly worse, and the TNR of the Asus Zenfone Selfie was quite bad, mainly because of the relatively large noise floor, as shown in Table 1. The TPR and TNR values of the smartphones with relatively large noise floors were improved, however, by developing their own classifier models. For instance, the TPR and TNR of the Asus Zenfone Selfie increased to 98.98% and 97.50%, respectively, when the classifier model was trained using the accelerations with the corresponding noise floor.

## 4. PGA Prediction

In this study, we tried to implement a single-station EEW approach on smartphones. The approach developed by Hsu et al. [21] using six features after a trigger to construct a PGA prediction model was implemented in this study. This PGA prediction model has thus far provided timely warnings during the 2016 Mw 6.4 Meinong, Taiwan earthquake [22] and the 2018 Mw 6.4 Hualian, Taiwan earthquake [23]. According to the relevant study by Hsu et al. [21], the PGA prediction model was constructed using a support vector machine algorithm. In this study, an ANN was utilized instead. The six features used were the peak acceleration (Pa), peak velocity (Pv), peak displacement (Pd), CAV, integral of square velocity (IV2), and predominant period (Tc) of the first N seconds after a trigger of the vertical acceleration component. For each length of N, a PGA prediction model was trained. The predominant period was calculated as

(1) $$T_{c}=2\pi /\sqrt{r},\;\mathrm{where}\;r=\int_{0}^{N}{\dot{u}}^{2}(t)dt/\int_{0}^{N}u^{2}(t)dt$$

where u(t) and $\dot{u}(t)$ are the vertical component of displacement and velocity time history of ground motion after *p*-wave arrival, respectively. Moreover, the integral of square velocity was calculated as

(2) $$IV2=\int_{0}^{N}{\dot{u}}^{2}(t)dt$$

**Figure 2:**
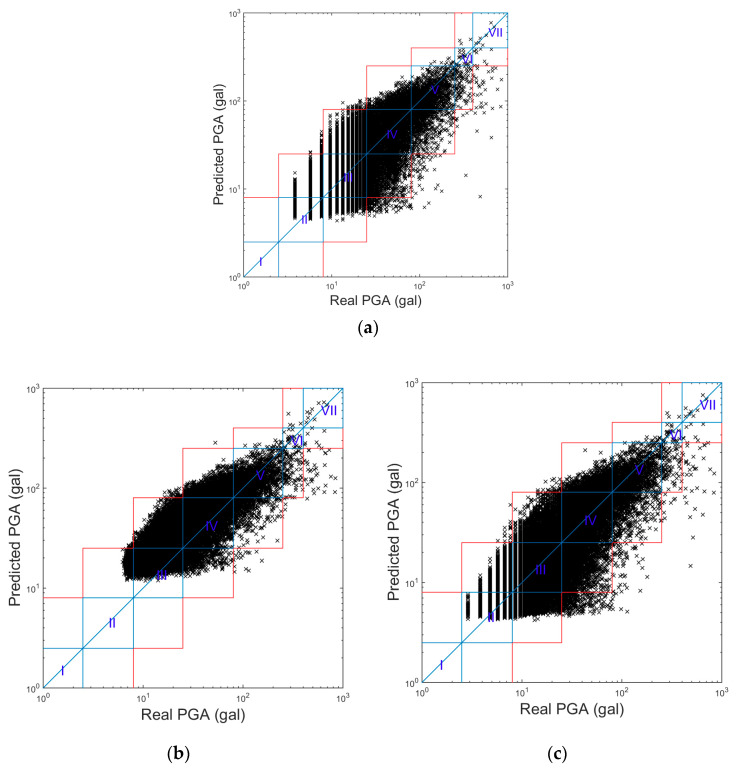

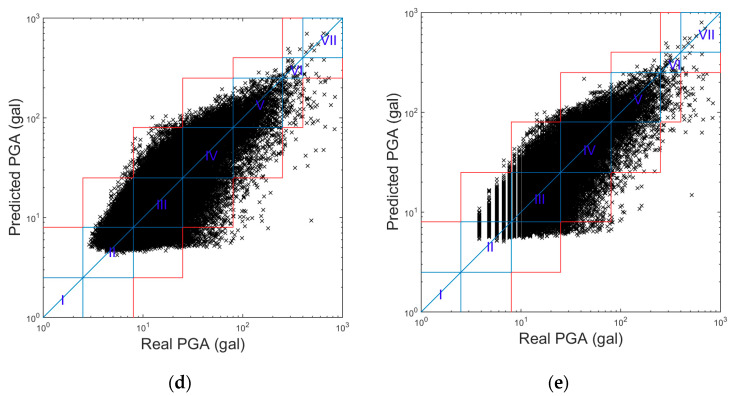
The comparison of the predicted PGA values of the typical 3 s prediction model (LG Stylus) with the real PGA values using smartphone (**a**) LG Stylus; (**b**) Asus ZenFone Selfie; (**c**) HTC Desire Eye; (**d**) Redmi Note4; (**e**) Huawei P3.

Again, 75%, 12.5%, and 12.5% of the same 10,000 pieces of earthquake data were used to train, validate, and test each PGA prediction model, respectively. After the zero-mean normalization of the original strong ground motion records was applied, the records were integrated once and twice to obtain velocity and displacement signals, respectively. The second-order 0.075 Hz high-pass Butterworth filter was applied to remove the low frequency drift after integration. The results of all the available earthquake data are shown in the following paragraphs of this section. The PGA prediction models were only constructed for the LG Stylus smartphone using earthquakes with corresponding resolution and noise floor values. A grid search was conducted to test different numbers of neurons and layers, and the best performance was achieved when the ANN had two hidden layers with 20 neurons with sigmoid activation function. The predicted PGA values of the typical 3 s prediction model are compared with the real PGA values for all the 77,994 pieces of earthquake data in Figure 2a. It seems that the predicted PGA values corresponded to the real PGA values relatively well on a logarithmic scale. The Roman numerals “I” to “VII” represent the seismic intensity scale in Taiwan, i.e., the CWB intensity [24]. The regions enclosed by the blue lines indicate cases for which the seismic intensity of the predicted PGA was the same as the real PGA, while the regions enclosed by the red lines indicate cases for which the seismic intensity of the predicted PGA was within ± one-scale value of the real one. Note that the resolution of the LG Stylus smartphone can be clearly observed in the figure when the real PGA is small. The minimum real PGA value was approximately 4 Gals, which corresponded to the noise floor of the LG Stylus smartphone.

**Figure 3:**
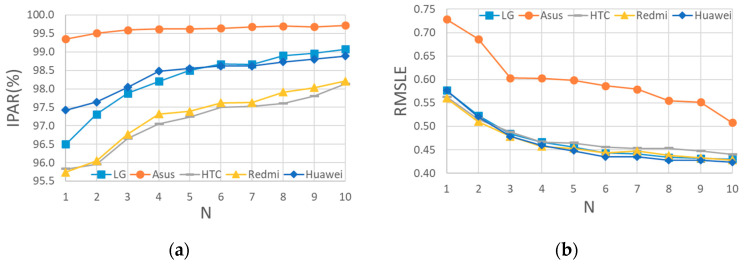
The (**a**) IPAR; and (**b**) RMSLE values of different N seconds for different smartphones.

We defined the intensity prediction accuracy ratio (IPAR) as the ratio at which the predicted intensity scale was located within a one-scale difference from the real intensity scale among all the considered earthquake data. Only the earthquake data with a measured intensity scale ≥4 or a predicted intensity scale ≥4 were considered. The IPAR values of the different N seconds of the LG Stylus smartphone are shown in Figure 3a. The IPAR was 96.5% at the first second, and then increased gradually to 99.1% at the 10th second. Furthermore, the root mean squared logarithmic error (RMSLE) of the predicted PGA values using the PGA prediction models of different N seconds are shown in Figure 3b. The RMSLE value was 0.577 at the first second, and then decreased gradually to 0.430 at the 10th second. Apparently, the accuracy of PGA prediction models for the LG Stylus smartphone was quite promising.

The above LG Stylus prediction models were applied to the other smartphones without any modification. The only difference, therefore, was in the acceleration quality of the smartphones themselves. Comparisons of the predicted and real PGA values of the typical 3 s prediction models of the different smartphones are shown in Figure 2b–d. The IPAR values and RMSLE values of these smartphones when using the LG Stylus prediction models for the different N values are also plotted in Figure 3a,b, respectively. In general, it seems that the application of the LG Stylus prediction models to the other smartphones without any modification was quite successful.

In terms of the RMSLE values, all the smartphones were quite comparable, with the exception of the ASUS ZenFone Selfie, whose RMSLE values were obviously larger than those of the other smartphones. Apparently, the predicted PGAs of ASUS ZenFone Selfie were biased to be larger than the real ones, as can be observed in Figure 2b. This was mainly because the noise floor of ASUS ZenFone Selfie is larger than that of the LG Stylus. Hence, the acceleration amplitude was larger when the smartphone was triggered, i.e., when the acceleration amplitude was constantly larger than the noise floor. If the *p*-wave features are calculated using larger amplitudes of acceleration, then intuitively the predicted PGA values based on these features tend to be larger.

In terms of the IPAR values, those of the Huawei P3 smartphone indicated similar performance to those of the LG Stylus smartphone. As for the HTC eye and Redmi Note4 smartphones, their IPAR values were lower, approximately 1% lower in general, than those of the LG Stylus smartphone. Meanwhile, the IPAR values of the ASUS ZenFone Selfie were larger than those of the LG Stylus. Again, these results were mainly affected by the noise floor values of the smartphones (which are listed in Table 1). The noise floors of the HTC eye and Redmi Note4 smartphones are a little bit lower than that of the LG Stylus. Hence, their predicted PGA values tended to be a little bit smaller. Therefore, more of the predicted PGA values were outside the one-scale difference range, especially in cases in which the real intensity scale value was IV and predicted intensity scale value was II, as can be observed in Figure 2c,d. As for the ASUS ZenFone Selfie, although the predicted PGA values tended to be larger, as mentioned above, the dispersion of the predicted PGA values for the ASUS ZenFone Selfie was actually smaller than that of the values for the LG Stylus. It thus seems that the accuracy of PGA prediction was higher when the accelerations after triggering at larger amplitudes were used. In contrast, the lead time before the arrival of large vibrations becomes shorter due to the use of later triggers. The effects of noise floor on the lead time will be discussed in the next section, in which the alert issuing performance of the proposed approach is analyzed. In addition, because the noise floor of the ASUS ZenFone Selfie is larger, the predicted intensity scale was always larger than scale II, as can be observed in Figure 2e. As a result, the IPAR values of the ASUS ZenFone Selfie were higher.

## 5. Alert Issuing Performance

The threshold for issuing an alert was set at intensity scale IV, and the confusion matrix of false positives (FP), false negatives (FN), true positives (TP), and true negatives (TN) is used here to detail the classification performance of the alerts. Note that only when an alert was issued before the threshold was reached was the alert classified as a TP. In addition, a tolerance range with a scale of ± 1 was used for defining whether a classification was acceptable or not (modified from [25]). Only if an alert was classified as a TP was the lead time meaningful and then used to analyze the lead time performance. The lead time was defined as the time difference between when a PGA prediction was first issued until the arrival of the PGA.

**Figure 4:**
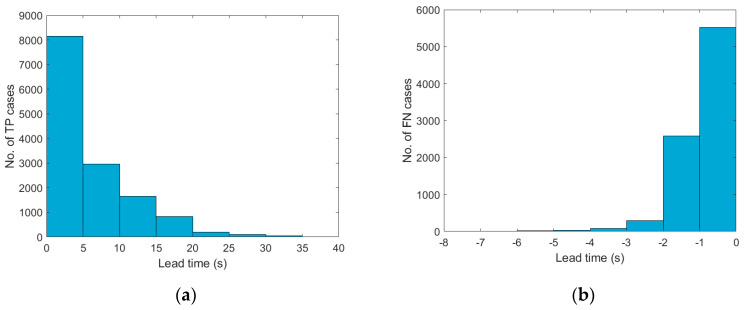
The distribution of the (**a**) TP cases; and (**b**) FN cases at different lead time ranges. (LG Stylus).

Among all the 77,994 pieces of earthquake data converted to the quality of the LG Stylus smartphone, 13,986, 8420, 446, and 54,955 were classified as TP, FN, FP, and TN, respectively. The distribution of the TP cases at different lead time ranges is summarized in Figure 4a. The lead time for approximately 58.3% of these TP cases was less than 5 s, while the lead time for approximately 21.3% was 5–10 s. The rest of these TP cases, approximately 20.4%, had more than 10 s of lead time. As for the FN cases, most of them occurred due to negative lead times (97.8% = 8420/8607), while only a few were because of an underestimation of the PGA. The distribution of these negative FN cases at different lead time ranges is summarized in Figure 4b, and apparently, the lead time for most of these cases (95.2%) was larger than (−)2 s. The number of FP cases was quite small, and all of them consisted of intensity scale II earthquakes predicted as scale IV earthquakes. The correct alert rate, CAR = TP/(TP + FP), which indicates the rate at which the alerts issued were actually correct, was 96.9%. The normalized TP rate, TPR = TP/(TP + FN), which indicates the rate at which an alert was issued before the arrival of the PGA when an earthquake exceeded the threshold, was 61.9%.

**Figure 5:**
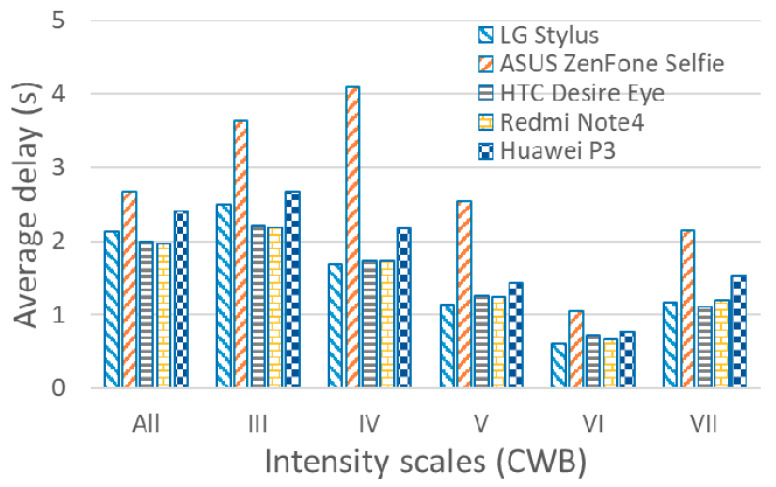
The average delay of lead time using different smartphones with 3 s prediction model for the earthquakes of intensity scales III to VII. The average delay of lead time of all the earthquakes is also shown (All).

In order to understand the effects of using the differing accelerations resulting from differing quality levels of smartphones on lead time, an on-site EEW approach conducted following the same procedures and using the same earthquakes with the original high quality of accelerations was employed as a reference. Here, without loss of generality, only the results using the typical 3 s prediction model are discussed for conciseness. The average delay of lead time (AD) using the different smartphones for the earthquakes of different intensity scales is summarized in Figure 5. The AD values of all the earthquakes using the different smartphones ranged between 1.96 and 2.68 s. In general, the larger the noise floor of the smartphone, the longer the AD was (marked as “All” in Figure 5). If the AD of the different intensity scales was considered, then generally the AD decreased with any increase of the intensity scale, except for the intensity scale values of VII. Nevertheless, the AD values when using the smartphones were approximately only 2 s if the noise floors of the smartphones were not too large, while the AD of the smartphone with the largest noise floor, i.e., the ASUS ZenFone Selfie, was approximately 0.5 s longer.

## 6. Shake Table Verification

Two strong motion datasets were employed to verify the performance of the proposed approach using smartphones during the shake table test. The shake table used was Moog’s Electric Motion Base MB-E-6DOF/24/1800KG in the National Center for Research on Earthquake Engineering, Taiwan. The first strong motion dataset was collected from worldwide earthquakes. Among the 50 significant earthquakes provided by the Center for Engineering Strong Motion Data (CESMD), a cooperative center established by the US Geological Survey (USGS) and the California Geological Survey (CGS), 10 strong motions were selected from these significant earthquakes that occurred in different regions. In addition, five more strong motions of the 1999 Chi-Chi earthquake, Taiwan, the 2018 Hualian earthquake, Taiwan, the 2008 Wenchun earthquake, China, the 1997 Northwest earthquake, China, and the 1995 Kobe earthquake, Japan were also included. In total, the worldwide earthquake dataset used consisted of 15 strong motions, as listed in Table 4.

**Figure 6:**
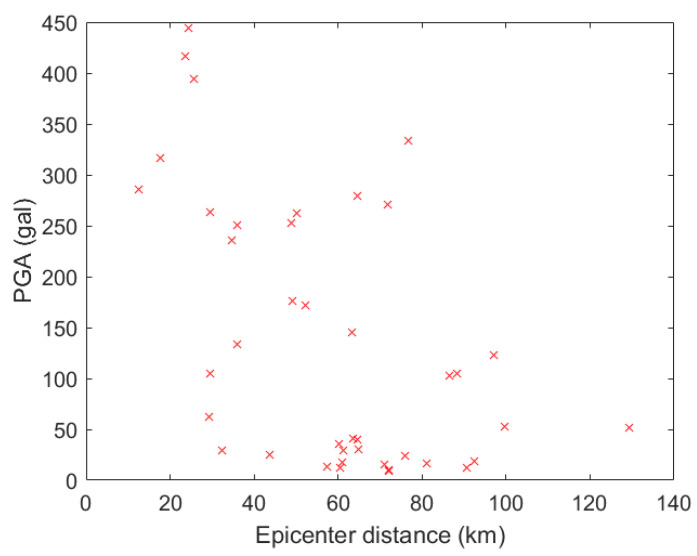
The distribution of the PGA and epicenter distance of the strong motions in the second dataset (Meinong earthquake).

Because the PGAs of most of the strong motions in the first dataset are quite large, the second of the two strong motion datasets was selected from the strong motions of the 2016 Meinong earthquake, Taiwan. The Meinong earthquake had a moment magnitude of 6.53 and a focal depth of 14.6 km and caused 117 deaths and 551 injuries, in addition to causing the collapse of six buildings and serious damage to 247 buildings. In total, 43 strong motions from small to large PGA values of the Meinong earthquake were selected. The distribution of the PGA values and the epicenter distance is shown in Figure 6.

**Figure 7:**
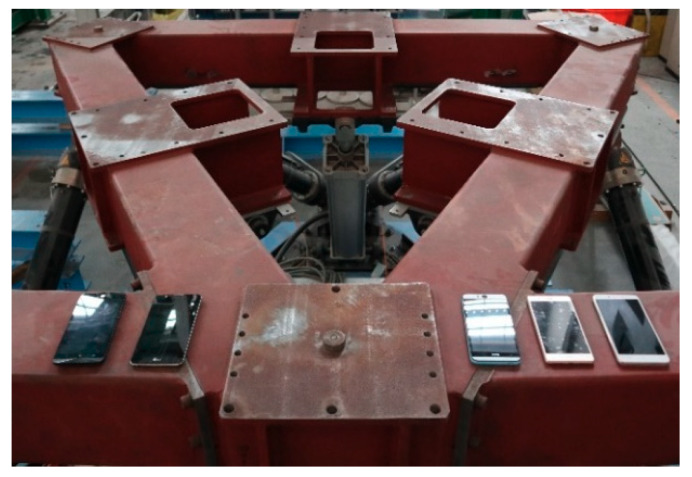
The smartphones mounted on the shake table.

**Figure 8:**
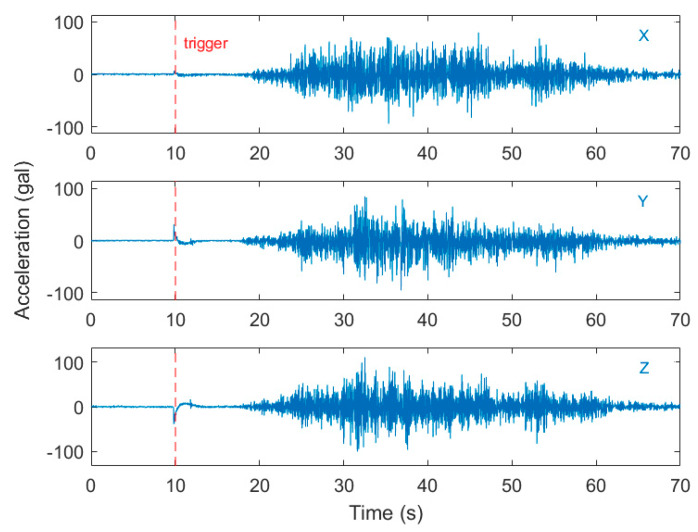
The typical case of false classification during the shake table test of the Samoa Islands earthquake in the first dataset (worldwide earthquakes). The trigger started some seconds before the arrival of the *p*-wave due to noise signals at rest. (vertical component).

**Figure 9:**
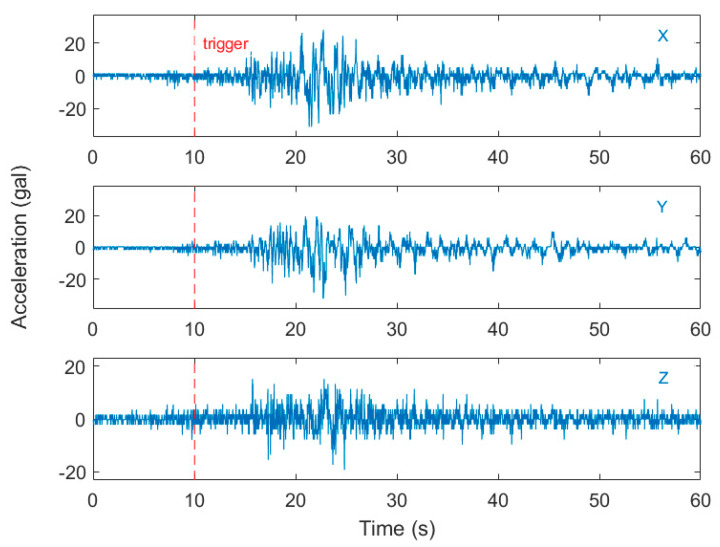
The typical case of false classification during the shake table test in the second dataset (Meinong earthquake). The PGA is only 27.8 Gals, i.e., just above the threshold to issue an alert (25 Gals).

The five smartphones were mounted on the shake table, as shown in Figure 7. The above-mentioned ANN classifiers and PGA prediction models using the features of different lengths of time after a trigger were embedded into the corresponding smartphones. For the first worldwide strong motion dataset, all 15 cases were classified correctly, except for one case being misclassified by the LG Stylus and two cases being misclassified by the Asus Zenfone Selfie. The reason for the false classifications was that the trigger started some seconds before the arrival of the *P*-wave due to the noise signals at rest. A typical case of false classification can be found in Figure 8. Once the smartphone was triggered, it took 10 s at least to start another trigger for the embedded program. Therefore, when the *P*-wave arrived, the program could not detect it and only recognized it as part of the trigger event that previously occurred. As for the second Meinong earthquake dataset, most of the 43 cases were classified correctly for all the smartphones. Only one to four cases with PGA values of only approximately 25 Gals were misclassified. This was mainly because the signal-to-noise ratios of these cases with small amplitudes were not high enough (see Figure 9).

**Figure 10:**
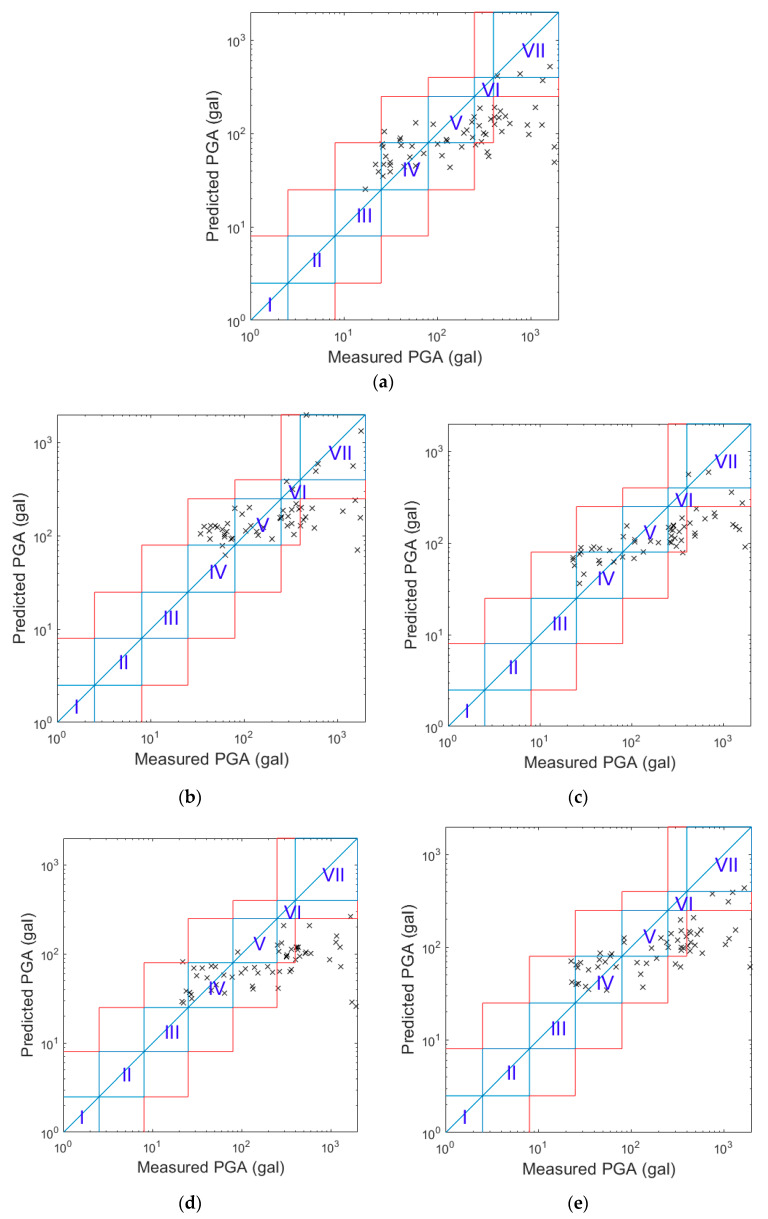
The comparison of the predicted PGA values with the real PGA values when the alerts were issued. (**a**) LG Stylus; (**b**) Asus ZenFone Selfie; (**c**) HTC Desire Eye; (**d**) Redmi Note4; (**e**) Huawei P3.

**Figure 11:**
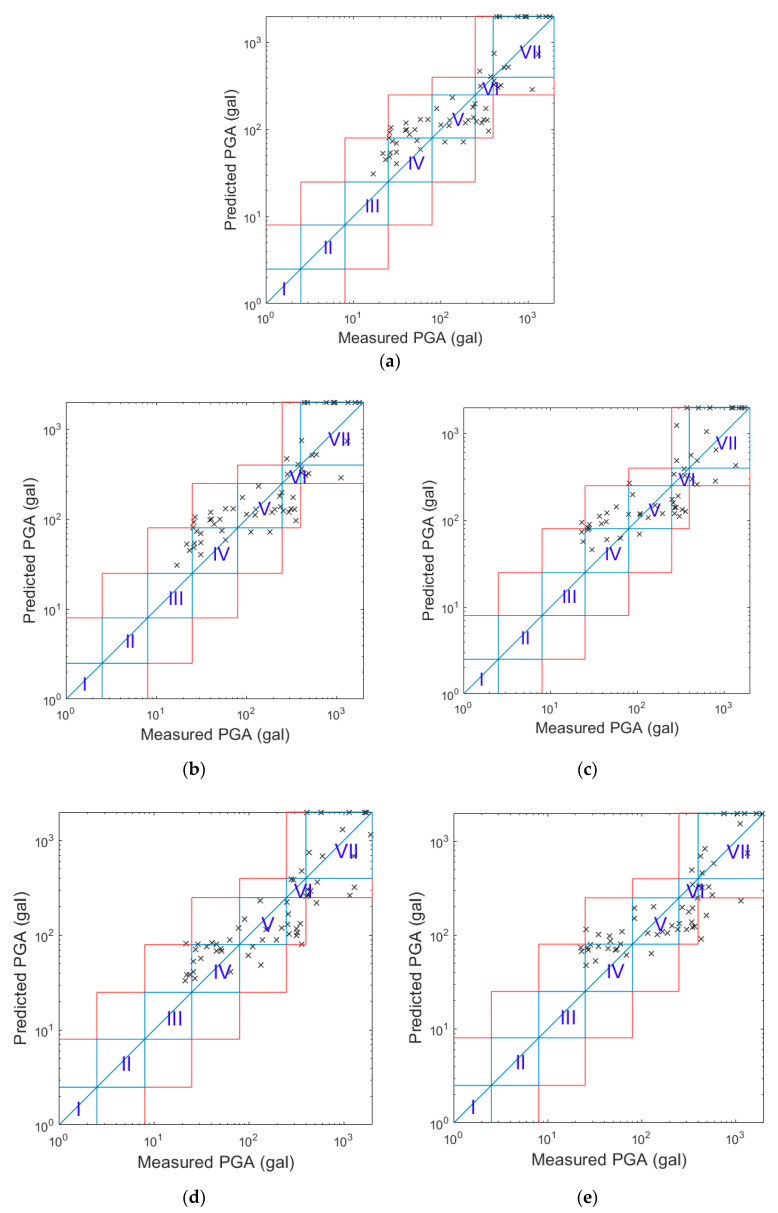
The comparison of the predicted PGA values with the real PGA values when *N* = 10 s. (**a**) LG Stylus; (**b**) Asus ZenFone Selfie; (**c**) HTC Desire Eye; (**d**) Redmi Note4; (**e**) Huawei P3.

After the strong motions were classified as an earthquake event, the program started to predict the PGA every second until 10 s after the trigger. Once the predicted PGA was larger than the threshold, i.e., 25 Gals, an alert could be issued. The comparisons of the predicted and real PGA values for both the worldwide and Meinong strong motion datasets when an alert was issued for the different smartphones are shown in Figure 10. The predicted PGAs were updated every second using the PGA prediction models for different values of N. The comparisons of the predicted and real PGA values when *N* = 10 s for the different smartphones are shown in Figure 11. Obviously, the predicted PGAs were much closer to the real ones when *N* = 10 s, since more information in terms of measured accelerations was used to predict the PGAs.

**Figure 12:**
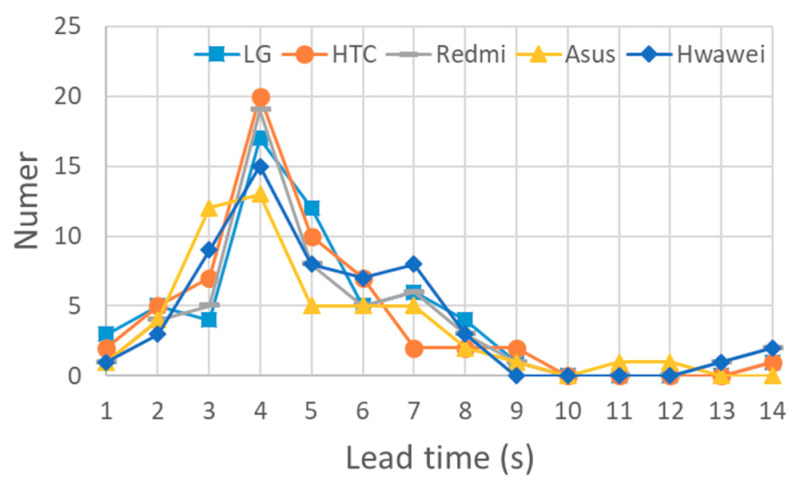
The distribution of lead times of both worldwide and Meinong strong motion datasets for different smartphones.

**Figure 13:**
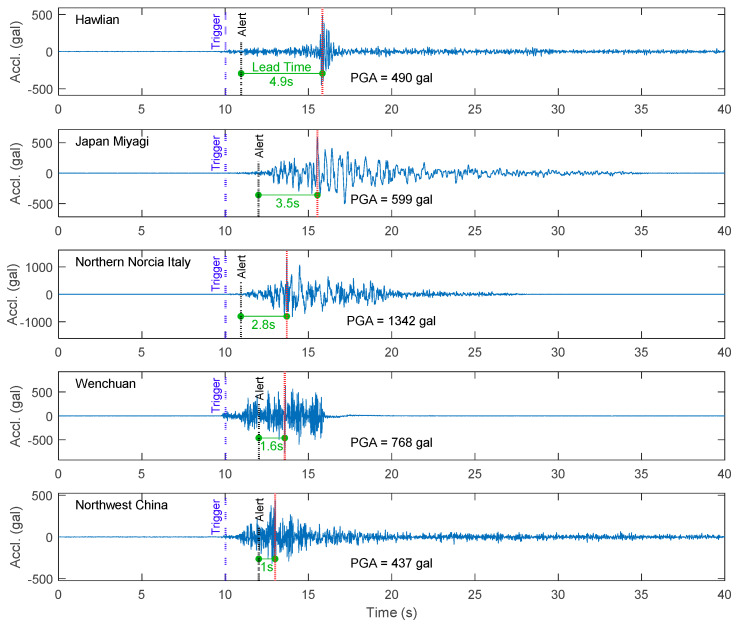
The typical five time histories with the shortest lead time for the worldwide earthquake dataset using the LG Stylus smartphone.

**Figure 14:**
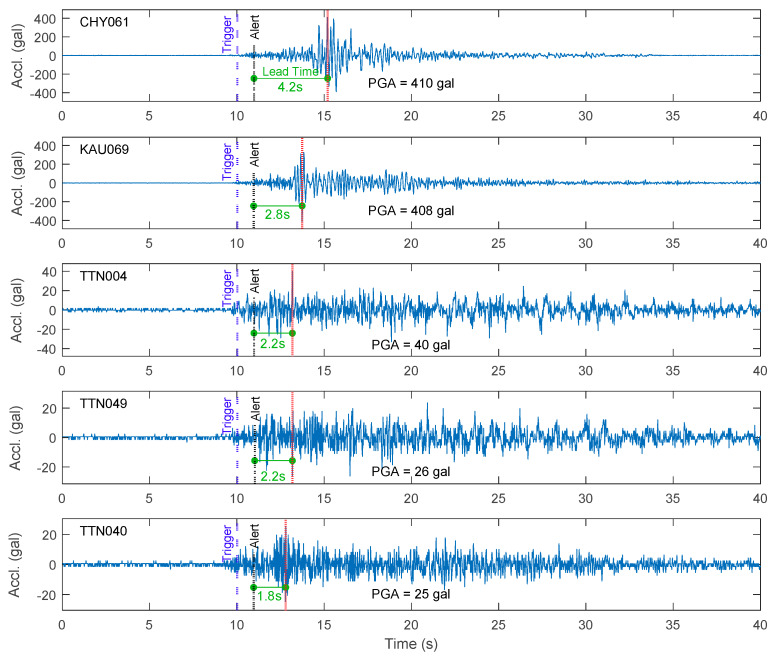
The typical five time histories with the shortest lead time for the Meinong earthquake dataset using the LG Stylus smartphone.

The lead time was calculated as the difference between the time at which an alert was issued and the arrival of the PGA. The distributions of lead times for both the worldwide and Meinong strong motion datasets for the different smartphones are summarized in Figure 12. In general, once the considered strong motions were classified as earthquake events, the proposed approach could provide 2–8 s of lead time for most of them. The typical five time histories with the shortest lead time for both the worldwide earthquake dataset and the Meinong earthquake dataset using the LG Stylus smartphone are plotted in Figure 13 and Figure 14, respectively. The time at the trigger, earthquake detection, alert issuing, and PGA occurrence of each time history is also marked to show in which phase the ground motion is predicted. Note that, for most of the earthquakes, once the earthquake was detected, the alert was issued at the same second. Again, the lead times for the Asus ZenFone Selfie tended to be a little bit shorter than the ones for the other smartphones due to its relatively high noise floor. Nevertheless, in conclusion, true positive (TP) alerts were provided by all the smartphones for all the considered strong motions classified as earthquake events.

## 7. Conclusions and Future Work

The possibility of providing on-site EEW using the measured accelerations of a single smartphone alone was investigated in this study. Once a trigger was classified as an earthquake event using the ANN classifier, the PGA was predicted using the ANN predictor. The classifier and predictor operated every second until 10 s after the trigger. An alert could be issued once the predicted PGA was larger than a threshold value at a certain second.

This study employed five smartphones of different brands and acceleration measurement quality to verify the proposed approach. The classifier and predictor were constructed based on the acceleration measurement quality of the LG Stylus smartphone, and could be applied to the other smartphones with similar or smaller noise floors after the accelerations of these smartphones were converted to the resolution of the LG Stylus smartphone.

Based on the results of large amounts of available non-earthquake and earthquake data, high percentages of TPRs and TNRs for all the smartphones with similar or smaller noise floors were obtained. Only some earthquakes with small PGA values were misclassified for these smartphones. As for the smartphone with a larger noise floor than the LG Stylus, i.e., the Asus Zenfone Selfie, the TNR was quite low. However, after a customized classifier based on the noise floor of that model was constructed, a TNR of a similar high level to those for the other smartphones was achieved.

Based on the results for a large amount of strong motion data, the accuracy levels of the predicted PGA values when using the LG Stylus model for all the smartphones were quite acceptable in general, despite the RMSLE and IPAR values being affected somewhat due to the different noise floors of the smartphones. Although the RMSLE was larger for the smartphone with a higher noise floor, i.e., the Asus Zenfone Selfie, the IPAR could also be higher.

Based on the results of a large amount of strong motion data for the LG Stylus smartphone, 96.9% of the issued alerts were correct and 61.9% of the earthquakes that exceeded the threshold resulted in an alert being issued before the arrival of the peak ground acceleration. Most of the false negative cases (97.8%) occurred because of a negative lead time. For the TP cases, less than 10 s of lead time could be provided for most of the cases. Compared to the results using original high-quality accelerations, the average delay of lead time using the smartphones was approximately only 2 s, except for the smartphone with the larger noise floor, i.e., the ASUS ZenFone Selfie, whose average delay was 0.5 s longer.

Based on the results of the shake table verification when using the program embedded into the smartphones, the performance of the proposed approach in terms of the earthquake classification and PGA prediction of the strong motions in the worldwide and Meinong datasets was quite promising. Moreover, true positive alerts were provided by all the smartphones for all the considered strong motions classified as earthquake events.

Nevertheless, substantial technological challenges remain for delivering earthquake early warning alerts using the proposed approach. For instance, false positive alerts could be issued because of non-earthquake events. In order to reduce the possibility of false positive alerts being issued due to non-earthquake events, a network detection algorithm is necessary. Therefore, the next step building upon this study will be to develop an app that can be downloaded to a smartphone by members of the public. With this app, only when enough smartphones within a certain area predict large PGA values will an alert be announced from the server to the smartphone users. In addition, the proposed approach uses the first several seconds of the *p*-wave records for earthquake detection and PGA prediction. If the smartphones are located at the upper floors of a building, the measured acceleration signals should also contain the structural response. Fortunately, at the very beginning of the measured acceleration signals, the structural response is usually not excited substantially; hence the ground excitation may not be contaminated by the structural response extensively. There are also other conditions may affect the performance of the proposed approach, e.g., smartphones placed on a soft object. In order to ascertain the effects on the performance of the proposed approach due to smartphone locations, further studies should be conducted in the future.

## Figures and Tables

**Table 1:** The acceleration quality and number of the ground motions of the smartphones.

Smartphone	Resolution (Gal)	Noise Floor (Gal)	Earthquake Data
LG Stylus	1.915	0.944	77,994
Asus ZenFone Selfie	0.239	2.048	36,476
HTC Desire Eye	1.000	0.783	73,250
Redmi Note4	0.120	0.867	76,387
Huawei P3	0.958	1.015	64,308

**Table 2:** The number of missed earthquakes among all 77,994 earthquake data of different PGA ranges after N seconds. The FPRs of all 49,094 non-earthquake cases after N seconds are also listed. (LG Stylus).

N	PGA Range (Gal)						Total	FPR(%)
	2.5~8	8~25	25~80	80~250	250~400	400~		
1	112	847	344	39	3	5	1350	3.20
2	13	182	81	4	0	1	281	4.39
3	2	49	22	1	0	0	74	5.11
4	2	21	8	1	0	0	32	5.53
5	1	7	4	0	0	0	12	5.87
6	1	5	4	0	0	0	10	6.13
7	1	2	3	0	0	0	6	6.34
8	1	2	1	0	0	0	4	6.50
9	1	1	1	0	0	0	3	6.63
10	1	1	1	0	0	0	3	6.76

**Table 3:** TPR and TNR of the 2 s ANN classifier of different smartphones using the LG classifier model. The TPR and TNR of the Asus ZenFone Selfie using the classifier model trained using the accelerations with corresponding noise floor are also listed in the brackets.

Smartphone	TPR	TNR
LG Stylus	99.09%	97.85%
Asus ZenFone Selfie	98.51% (98.98%)	61.68% (97.50%)
HTC Desire Eye	98.93%	97.76%
Redmi Note4	99.70%	99.79%
Huawei P3	99.06%	99.66%

**Table 4:** List of the 15 ground motions of worldwide earthquake dataset.

Earthquake	Region	Station	Epicentral Distance (km)	Date	Mw	PGA (gal)
Northridge	US/CA	Cedar Hill Nursery A	16.7	1994/01/17	6.7	1744.5
Kobe	Japan	Nishi-Akashi	38	1995/01/17	7.3	605.28
Northwest China	China	CSB station 19001	27.7	1997/04/11	6.1	376.56
Chi Chi	Taiwan	TCU084	18	1999/09/21	7.6	681.62
Hector Mine	US/CA	Mill Creek; Ranger Station	91.27	1999/10/16	7.1	59.45
Wenchuan	China	051WCW	14	2008/05/12	8	957.7
L’Aquila	Italy	L’AQUILA-V. ATERNO-F. ATERNO	4.6	2009/04/06	6.3	393.4
Samoa Islands	Tonga	Afiamalu, Samoa	179	2009/09/29	8	103
Miyagi	Japan	Shiogama-MYG012	142	2011/04/07	7.1	1447.02
Northern Norcia	Italy	Forca Canapine	11.7	2016/10/30	6.5	910.37
Amberley	New Zealand	Molesworth Station	70	2016/11/13	7.8	312.7
Valparaiso	Chile	Torpederas	39	2017/04/24	6.9	889
Raboso	Mexico	Unam-Mexico	116.4	2017/09/19	7.1	53.7
Hawlian	Taiwan	ENA	35.63	2018/02/06	6.2	428.31
Anchorage	US/AK	Rabbit Creek AK USA	46	2018/11/30	7	651.5
